# Effect of the rearing system on the color of four muscles of suckling kids

**DOI:** 10.1002/fsn3.994

**Published:** 2019-03-20

**Authors:** Guillermo Ripoll, María Jesús Alcalde, Anastasio Argüello, María de Guía Córdoba, Begoña Panea

**Affiliations:** ^1^ Instituto Agroalimentario de Aragón—IA2—(CITA‐Universidad de Zaragoza) Zaragoza Spain; ^2^ Animal Production and Health Unit Centro de Investigación y Tecnologia Agroalimentaria de Aragón Zaragoza Spain; ^3^ Department of Agroforestry Science Universidad de Sevilla Sevilla Spain; ^4^ Universidad de Las Palmas de Gran Canaria Las Palmas Spain; ^5^ Department of Food and Nutrition, Instituto Universitario de Investigación de Recursos Agrarios (INURA) Escuela de Ingeniería Agrarias Universidad de Extremadura Badajoz Spain

**Keywords:** biceps femoris, chop, milk replacer, semimembranosus, semitendinosus

## Abstract

Most suckling kids are raised on farms for cheese production, and many goat farmers rear kids with milk replacers. The aim of this work was to study the influence of the use of milk replacers on the color of four muscles. A total of 246 suckling kids of eight breeds were slaughtered to achieve carcasses of 5 kg. The color of the *biceps femoris*, *semimembranosus*, *semitendinosus,* and *longissimus thoracis* muscles was measured with a spectrophotometer, and CIELab coordinates were registered. In addition, the pH of *longissimus thoracis* was measured. The effect of the rearing system (RS) on the color of the studied muscles is strongly modulated by breed. In general terms, there are two groups of kids according to the color of meat. The first group has great lightness and hue angle including Malagueña, Palmera, and Tinerfeña fed natural and artificial milk. The second group with great redness includes Retinta, Payoya, and Verata fed natural and artificial milk together with Florida fed natural milk and Cabra del Guadarrama fed milk replacers. Hence, farms should consider selecting a breed and RS together. Most of the kid meat with high pH comes from kids raised on milk replacers. Because artificial RSs use very early weaning, which might induce a high pH and dark meat, two artificial rearing strategies can be proposed. The first strategy is to choose less sensitive breeds that produce meat with a normal pH. The second strategy is to restrict suckling of natural milk but minimize separation from the mother.

## 1 INTRODUCTION

Spain produces 10.9% of kid meat in the European Union, and 80% of this kid meat originates from the light suckling kid category (*cabrito*) (MAPAMA, 2016). Goat milk is a valuable product. Therefore, some goat farmers remove kids from their dams at a very young age and rear them with milk replacers to produce cheese from the mother's milk. When kid goats are reared with their dams, the quantity and quality of milk available for cheese production may change. Hence, milk from Florida, with no suckling kids, has more fat, protein, and nonfat dry extract than milk from dams’ suckling kids (Delgado‐Pertíñez, Guzmán‐Guerrero, Mena, et al., 2009), although other breeds only show a tendency to increase fat and protein in milk from dams with no suckling kids (Delgado‐Pertíñez, Guzmán‐Guerrero, Caravaca, et al., 2009). However, some farmers still rear kids with their dams because it is believed that kid meat is more tender when they are reared with natural milk than artificial suckling systems (Argüello, Castro, Capote, & Solomon, 2005). On the other hand, the leg chops of kids reared with milk replacers are preferred by consumers because the meat is paler and dull, having lower a* and $C_{\mathrm{ab}}^{*}$ values. Additionally, the purchase intention during the time of display is greater for kids reared with natural milk than that for kids reared with milk replacers (Ripoll, Alcalde, Argüello, Córdoba, & Panea, 2018). Although the *longissimus thoracis et lumborum* (LTL) is the most common muscle used in studies focused on suckling kid meat color (Bañón, Vila, Price, Ferrandini, & Garrido, 2006; Bonvillani et al., 2010; Dhanda, Taylor, & Murray, 2003; Ekiz, Ozcan, Yilmaz, Tölü, & Savas, 2010; Teixeira, Jimenez‐Badillo, & Rodriguez, 2011; Todaro et al., 2004), other studies have measured the color of other muscles, such as *semitendinosus* or *triceps brachii* (Argüello et al., 2005; Marichal, Castro, Capote, Zamorano, & Arguello, 2003; Zurita‐Herrera, Delgado, Argüello, Camacho, & Germano, 2013). Kid meat is often sold as rib and leg chops. The largest muscle by area in rib chops is the LTL, but leg chops provide other muscles to consumers than the LTL, which are also important for the purchase decision (Ripoll et al., 2018). Muscles from the leg and LTL have different functions, different metabolism patterns, and different proportions of muscle fiber types, which are correlated with color (Lee, Joo, & Ryu, 2010). Hence, the use of natural milk or milk replacers could affect the color of the muscles differently. Therefore, the aim of this work was to study the influence of the use of milk replacers on the color of four muscles of eight breeds of suckling kids.

## 2 MATERIALS AND METHODS

### 2.1 Animals

All procedures were conducted according to the guidelines of Directive 2010/63/EU on the protection of animals used for experimental and other scientific purposes (EU, 2010).

Suckling male kids of eight goat breeds (Florida, FL; Cabra del Guadarrama, GU; Majorera, MA; Palmera, PL; Payoya, PY; Retinta, RE; Tinerfeña, TI; Verata, VE) were reared in two or three farms per breed in their respective local areas. Animals were all born from single parturitions. Half of these animals were raised with milk replacers (MR), and the other half were raised with natural milk from dams (NM). Kids reared on the MR RS were fed colostrum for the first 2 days and had free access to the milk replacer 24 hr per day, which was suckled from a teat connected to a unit for feeding a liquid diet. Commercial milk replacers were reconstituted at 17% (w/v) and given warm (40°C). The main ingredients were skimmed milk (≈60%) and whey. The chemical composition of milk replacers was as follows: total fat 25 ± 0.6%, crude protein 24 ± 0.5%, crude cellulose 0.1 ± 0.0%, ash 7 ± 0.6%, Ca 0.8 ± 0.1%, Na 0.5 ± 0.2%, P 0.7 ± 0.0%, Fe 36 ± 4.0 mg/kg, Cu 3 ± 1.7 mg/kg, Zn 52 ± 18.8 mg/kg, Mn 42 ± 14.4 mg/kg, I 0.22 ± 0.06 mg/kg, Se 0.1 ± 0.06 mg/kg, and BHT 65 ± 30 ppm. Kids reared on the NM rearing system suckled directly from dams with no additional feedstuff. At night, they were housed with their dams in a stable. Kids from both RSs had no access to concentrates, hay, forages, or other supplements.

The numbers of kids used are shown in Table 1. A total of 246 kids were slaughtered at a live weight of 8.47 ± 0.077 kg. Standard commercial procedures according to the European guidelines for the protection of animals at the time of killing (EU, 2009) were followed. Head‐only electrical stunning was applied (1.00 A) to kids, and they were then exsanguinated and dressed. Thereafter, hot carcasses, including the head and kidneys, were weighed, achieving a hot carcass weight (HCW) of 4.97 ± 0.061 kg. Afterward, carcasses were hung by the Achilles tendon and chilled for 24 hr at 4°C.

**Table 1:** Color and pH of the *longissimus thoracis* muscle of kids reared with a milk replacer (MR) or natural milk from their dams (NM)

B	RS	*n*	pH	L*	a*	b*	*h_ab_*	$C_{\mathrm{ab}}^{*}$
FL	MR	15	5.54^g^	58.75^a^	−1.20^g^	12.42^bcd^	95.70^a^	12.34^cde^
	NM	15	5.49^g^	55.69^abcd^	0.55^fg^	13.08^b^	88.06^b^	12.91^cde^
GU	MR	15	5.75^de^	50.84^defg^	4.31^d^	8.28^ef^	62.73^f^	9.48^f^
	NM	16	5.71^def^	48.66^fg^	7.37^c^	9.00^e^	50.80^g^	12.06^cde^
MA	MR	16	5.84^cd^	52.5^cdefg^	9.40^c^	7.23^fg^	39.82^h^	11.83^de^
	NM	16	5.86^cd^	54.42^abcdef^	14.95^a^	7.33^fg^	25.53^jk^	16.40^a^
PL	MR	15	6.38^a^	58.61^ab^	14.03^ab^	5.78^h^	20.97^k^	14.66^abc^
	NM	16	5.97^bc^	54.81^abcde^	12.02^b^	6.08^gh^	27.29^ij^	13.24^cde^
PY	MR	16	5.78^dc^	54.45^abcde^	2.35^def^	13.46^b^	80.83^cd^	13.79^bcd^
	NM	14	5.64^efg^	49.15^fg^	1.54^f^	11.25^d^	83.07^bc^	11.52^de^
RE	MR	15	5.54^fg^	55.85^abc^	3.95^de^	15.22^a^	75.62^de^	15.82^ab^
	NM	15	5.47^g^	53.33^bcdefg^	3.86^de^	13.26^b^	74.26^e^	13.83^bcd^
TI	MR	16	6.05^b^	47.94^g^	12.33^b^	7.18^fg^	31.51^i^	14.09^bcd^
	NM	16	5.84^cd^	56.99^abc^	12.27^b^	6.38^gh^	27.98^ij^	13.70^cde^
VE	MR	15	5.75^de^	50.32^efg^	0.73^fg^	11.77^cd^	86.12^bc^	11.60^de^
	NM	15	5.83^cd^	53.77^bcdef^	1.89^ef^	13.01^bc^	82.08^bc^	13.18^cde^
	*SE*		0.037	1.587	0.712	0.452	2.145	0.705
	B		0.0001	0.0001	0.0001	0.0001	0.0001	0.0001
	RS		0.0001	0.70	0.003	0.28	0.0001	0.25
	B × RS		0.0001	0.0001	0.0001	0.0001	0.0001	0.0001

Notes

The least square means were adjusted for a hot carcass weight of 4.97 kg. Different superscripts in the same column indicate significant differences (*p* < 0.05).

B: breed; FL: Florida; GU: Cabra del Guadarrama; MA: Majorera; PL: Palmera; PY: Payoya; RE: Retinta; RS: rearing system; *SE*, standard error; TI: Tinerfeña; VE: Verata.

### 2.2 Carcass sampling

After carcass chilling, the LTs of the right half of the carcasses were extracted, vacuum‐packed, and aged for 3 days. Afterward, the *longissimus thoracis* muscle (LT) was extracted from the bag, a layer of muscle was retired to allow 1 hr of blooming, and the color was measured. Then, pH was measured with a pH meter equipped with a Crison 507 penetrating electrode (Crison Instruments S.A., Barcelona, Spain).

The right hind leg was vacuum‐packed and stored at −20°C. Then, a chop of each frozen leg was sliced and thawed overnight at 4°C in darkness. The *biceps femoris* (BF), *semimembranosus* (SM), and *semitendinosus* (ST) muscles of the leg were chosen (Popesko, 1977) because they are the muscles that represent most of the area of the chop, and the color was measured.

### 2.3 Instrumental color

The muscle colors were measured using a Minolta CM‐2006d Spectrophotometer (Konica Minolta Holdings, Inc., Osaka, Japan) in CIEL*a*b* space (CIE, 1986) with the specular component included, 0% UV, an observer angle of 10° and zero, and white calibration. The integrating sphere had a 52 mm diameter, and the measurement area (diameter of 8 mm) was covered with a CM‐A149 dust cover (Konica Minolta Holdings, Inc.). The illuminant used was D65. The spectrophotometer was rotated 90° on the horizontal plane before each reading, and the mean of three readings was used for analysis. The lightness (L*), redness (a*), and yellowness (b*) indexes were recorded using the SpectraMagic NX software (Minolta Co. Ltd., Osaka, Japan), and the hue angle $\left(h_{\mathrm{ab}}=\tan^{-1}\left(\frac{b^{*}}{a^{*}}\right)\cdot \frac{180^{\circ }}{\pi }\right)$ and chroma $\left(C_{\mathrm{ab}}^{*}=\sqrt{{\left(a^{*}\right)}^{2}+{\left(b^{*}\right)}^{2}}\right)$ were calculated.

### 2.4 Statistical analysis

All statistics were calculated using the XLSTAT statistical package v.3.05 (Addinsoft, USA). The studied variables were analyzed using ANCOVA with the breed (B), RS, and their interaction as fixed effects and the HCW as a covariate. The least square means were estimated, and differences between means were tested with the Bonferroni test at a 0.05 level of significance.

Principal component analysis (PCA) was performed with the color variables of the four muscles studied. PCA was performed to determine the number of independent variables that account for most of the data variation. Variables with a factorial load >0.7 were retained, and PCA was repeated. The retained variables were the L*, a*, and *h_ab_* of the four muscles. The KMO test value was 0.84, and the Bartlett test of sphericity was significant (*p* < 0.0001). A varimax rotation was applied to redistribute the variance among factors.

Kids were clustered together based on their pH by *k*‐means clustering. The number of clusters was selected to identify significant pH differences among all clusters and to avoid clusters formed by 10 or fewer observations. Then, ANCOVA was carried out for the pH and color variables, with the pH cluster as fixed effect and HCW as a covariate. A Bonferroni test was used to compare means, with a significance of *p* < 0.05. The independence between the RS and cluster of pH was tested with the chi‐square test.

## 3 RESULTS

### 3.1 pH of the longissimus thoracis

The pH ranged from 5.49 to 6.38 (Table 1). There was a significant interaction between breed and RS (*p* < 0.001). The RS did not modify the pH in most of the breeds (*p* > 0.05). However, Palmera, Payoya, and Tinerfeña kids reared with milk replacers had a higher pH than that of kids reared with natural milk (*p* < 0.05).

### 3.2 Instrumental color of the muscles

The color of LT is shown in Table 1. All of the color variables were affected by the interaction between the breed and RS (*p* < 0.001). The use of milk replacers increased the L* of Payoya (*p* < 0.05) and decreased L* of Tinerfeña (*p* < 0.05), but the other breeds were not affected (*p* > 0.05). There was a large range of *h_ab_* values, from 20.97 to 95.70. Most of the breeds were not affected by the RS, but Florida, Cabra del Guadarrama, and Malagueña kids had increased *h_ab_*values when fed with MR (*p* < 0.05).

The color of the muscle BF is shown in Table 2. All of the color variables except b* were affected by the interaction between the breed and RS (*p* < 0.05), but the breed and RS separately affected b* (*p* < 0.01). Guadarrama and Majorera fed NM had greater a* and $C_{\mathrm{ab}}^{*}$ values than those of their counterparts fed MR. However, in the other breeds, no differences were found between RSs for any of the rest of the considered variables (*p* > 0.05).

**Table 2:** Color of the *biceps femoris *muscle from kids reared with a milk replacer (MR) or natural milk from their dams (NM)

B	RS	L*	a*	b*	*h_ab_*	$C_{\mathrm{ab}}^{*}$
FL	MR	46.83^a^	4.18^e^	7.76^ab^	62.05^a^	8.86^e^
	NM	44.81^ab^	6.35^cde^	7.94^ab^	51.14^ab^	10.33^cde^
GU	MR	42.80^bcd^	7.31^cd^	7.37^abc^	45.08^bc^	10.50^cde^
	NM	39.99^d^	10.62^a^	7.49^abc^	35.70^c^	13.29^ab^
MA	MR	45.62^ab^	5.28^de^	7.97^ab^	56.33^ab^	9.79^de^
	NM	44.42^ab^	7.60^bc^	8.97^a^	49.31^ab^	11.86^abc^
PL	MR	45.58^ab^	5.90^cde^	8.09^ab^	54.64^ab^	10.25^cde^
	NM	45.55^ab^	6.82^cd^	8.73^ab^	52.57^ab^	11.21^bcd^
PY	MR	40.44^cd^	7.89^bc^	5.55^c^	35.35^c^	9.78^de^
	NM	43.03^abcd^	7.09^cd^	6.91^bc^	44.40^bc^	10.18^cde^
RE	MR	46.25^ab^	7.50^bc^	9.23^a^	50.67^ab^	11.95^abc^
	NM	42.67^bcd^	9.82^ab^	9.00^a^	43.04^bc^	13.43^a^
TI	MR	44.82^ab^	6.06^cde^	7.41^abc^	51.70^ab^	9.80^de^
	NM	45.48^ab^	6.51^cd^	8.91^a^	54.36^ab^	11.23^bcd^
VE	MR	43.98^abc^	7.09^cd^	8.21^ab^	49.12^b^	10.88^cd^
	NM	46.01^ab^	6.31^cde^	8.69^ab^	53.86^ab^	10.84^cd^
	*SE*	0.774	0.457	0.395	2.586	0.386
	B	0.0001	0.0001	0.0001	0.0001	0.0001
	RS	0.16	0.0001	0.002	0.051	0.0001
	B × RS	0.001	0.0001	0.290	0.0001	0.015

Notes

The least square means were adjusted for a hot carcass weight of 4.97 kg.

Different superscripts in the same column indicate significant differences (*p* < 0.05).

B: breed; FL: Florida; GU: Cabra del Guadarrama; MA: Majorera; PL: Palmera; PY: Payoya; RE:Retinta; RS: rearing system; *SE*: standard error; TI: Tinerfeña; VE: Verata.

The color of the muscle SM is shown in Table 3. L*, b*, and *h_ab_* were only affected by breed (*p* < 0.001), and a* and $C_{\mathrm{ab}}^{*}$ were influenced by the interaction between the RS and breed (*p* < 0.05). Therefore, the number of color variables and also the level of significance of the affected variables were lower than those of the other muscles. MA had a greater a* value when fed NM than when fed MR (*p* > 0.05). The use of NM tended to increase $C_{\mathrm{ab}}^{*}$, especially for GU and MA, although the differences between RSs were not significant (*p* > 0.05).

**Table 3:** Color of the *semimembranosus *muscle from kids reared with a milk replacer (MR) or natural milk from their dams (NM)

B	RS	L*	a*	b*	*h_ab_*	$C_{\mathrm{ab}}^{*}$
FL	MR	47.56^a^	4.19^e^	7.26^abc^	59.45^ab^	8.37^f^
	NM	47.40^ab^	5.52^cde^	6.33^bc^	65.58^a^	8.57^ef^
GU	MR	43.37^ab^	7.23^abcd^	7.26^abc^	44.70^ab^	10.38^abcde^
	NM	41.70^ab^	9.60^a^	7.27^abc^	40.08^b^	12.29^a^
MA	MR	44.79^ab^	5.28^de^	7.32^abc^	54.05^ab^	9.27^def^
	NM	44.23^ab^	7.36^abc^	7.98^ab^	45.69^ab^	11.02^abcd^
PL	MR	44.82^ab^	5.87^cde^	7.52^abc^	51.64^ab^	9.80^cdef^
	NM	44.59^ab^	6.75^bcd^	8.07^ab^	49.24^ab^	10.78^abcd^
PY	MR	41.69^b^	7.18^bcd^	5.77^c^	38.89^b^	9.40^def^
	NM	42.89^ab^	6.82^bcd^	6.51^bc^	44.50^ab^	9.64^cdef^
RE	MR	45.98^ab^	7.20^abcd^	8.94^a^	50.46^ab^	11.56^abc^
	NM	43.32^ab^	8.82^ab^	7.94^ab^	43.87^ab^	12.03^ab^
TI	MR	45.03^ab^	5.68^cde^	7.07^abc^	50.85^ab^	9.31^def^
	NM	44.52^ab^	6.33^cde^	8.29^ab^	52.64^ab^	10.69^abcde^
VE	MR	44.79^ab^	6.46^cd^	7.82^ab^	51.55^ab^	10.15^bcde^
	NM	46.16^ab^	6.02^cde^	7.84^ab^	52.93^ab^	9.97^cdef^
	*SE*	1.186	0.445	0.407	5.099	0.394
	B	0.0001	0.0001	0.0001	0.001	0.0001
	RS	0.51	0.0001	0.44	0.73	0.0001
	B × RS	0.80	0.012	0.053	0.73	0.042

Notes

The least square means were adjusted for a hot carcass weight of 4.97 kg. Different superscripts in the same column indicate significant differences (*p* < 0.05).

B: breed; FL: Florida; GU: Cabra del Guadarrama; MA: Majorera; PL: Palmera; PY: Payoya; RE:Retinta; RS: rearing system; *SE*: standard error; TI: Tinerfeña; VE: Verata.

The color of the muscle ST is shown in Table 4. All of the color variables except $C_{\mathrm{ab}}^{*}$ were affected by the interaction between the breed and RS (*p* < 0.05). In general terms, the use of MR increased the L* and *h_ab_* values and decreased the a* and $C_{\mathrm{ab}}^{*}$ values. However, the use of MR conversely affected the color of Payoya meat. Florida and Majorera fed MR had the highest L* value, and Guadarrama fed NM had the lowest L* value (*p* < 0.05). Florida and Majorera reared with MR had the highest *h_ab _*value and the lowest $C_{\mathrm{ab}}^{*}$ value (*p* < 0.05). On the other hand, Guadarrama fed NM had the lowest *h_ab _*value (*p* < 0.05). Florida fed MR and Guadarrama fed NM had the lowest and the highest $C_{\mathrm{ab}}^{*}$ values (*p* < 0.05), respectively.

**Table 4:** Color of the *semitendinosus *muscle from kids reared with a milk replacer (MR) or natural milk from their dams (NM)

B	RS	L*	a*	b*	*h_ab_*	$C_{\mathrm{ab}}^{*}$
FL	MR	49.71^a^	3.36^g^	8.54^cd^	68.43^a^	9.19^e^
	NM	48.29^abc^	5.05^defg^	8.82^bcd^	60.20^abc^	10.25^de^
GU	MR	44.89^bcde^	7.14^abcde^	8.76^bcd^	51.30^cdef^	11.42^abcd^
	NM	41.64^e^	9.73^a^	7.86^d^	39.93^f^	12.70^a^
MA	MR	49.40^a^	4.28^fg^	9.83^abc^	67.23^a^	10.96^cd^
	NM	47.15^abcd^	6.22^bcdef^	10.18^ab^	58.96^abcd^	12.09^abcd^
PL	MR	48.11^abcd^	5.23^bcde^	9.29^bcd^	61.04^abc^	10.87^cde^
	NM	47.36^abcd^	6.72^bcde^	9.73^abc^	56.48^abcde^	12.04^abcd^
PY	MR	44.15^de^	7.68^abcd^	7.95^d^	46.11^ef^	11.12^abcd^
	NM	46.27^abcd^	6.26^bcdef^	8.44^cd^	54.57^bcde^	10.87^cd^
RE	MR	46.69^bcd^	6.78^bcde^	9.65^abc^	54.72^bcde^	11.85^abcd^
	NM	44.71^bcde^	8.67^ab^	8.53^cd^	45.00^ef^	12.23^abc^
TI	MR	48.74^ab^	4.64^efg^	9.77^abc^	65.38^ab^	11.02^abcd^
	NM	48.36^ab^	5.95^bcdef^	10.77^a^	61.76^abc^	12.51^ab^
VE	MR	44.84^bcde^	7.38^abcde^	7.94^d^	47.38^def^	10.96^bcd^
	NM	44.52^cde^	7.69^abc^	8.19^d^	47.23^def^	11.40^abcd^
	*SE*	0.778	0.512	0.299	2.577	0.320
	B	0.0001	0.0001	0.0001	0.0001	0.0001
	RS	0.010	0.0001	0.51	0.000	0.0001
	B × RS	0.041	0.006	0.009	0.005	0.08

Note

The least square means were adjusted for a hot carcass weight of 4.97 kg. Different superscripts in the same column indicate significant differences (*p* < 0.05).

B: breed; FL: Florida; GU: Cabra del Guadarrama; MA: Majorera; PL: Palmera; PY: Payoya; RE:Retinta; RS: rearing system; *SE*: standard error; TI: Tinerfeña; VE: Verata.

**Figure 1:**
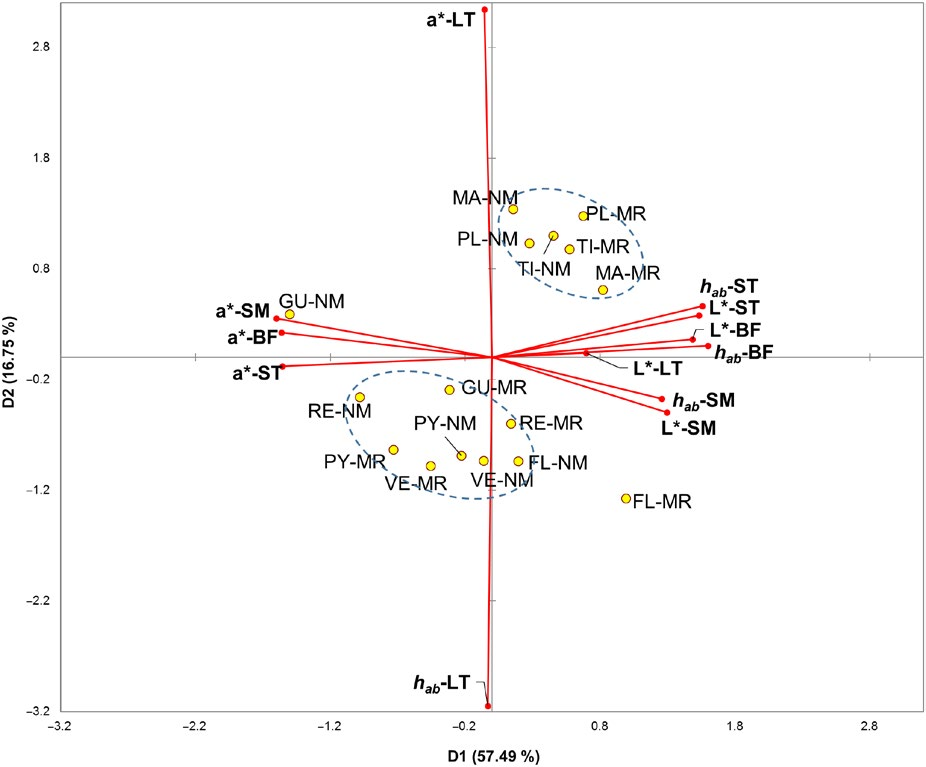
Biplot of principal component analysis of the instrumental color of four muscles from kids reared with a milk replacer (MR) or natural milk (NM) from their dams. BF: *biceps femoris*; FL: Florida; GU: Cabra del Guadarrama; LT: *longissimus thoracis; *MA: Majorera; MR: milk replacer; NM: natural milk; PL: Palmera; PY: Payoya; RE: Retinta; *SE*: standard error; SM: *semimembranosus*; ST: *semitendinosus*; TI: Tinerfeña; VE: Verata

Figure 1 shows a biplot of the PCA of the color of the four studied muscles. The first component explained 57.5% of the data variability. This component included a* of the three leg muscles in a negative way, but included the L* of the four muscles and *h_ab_* of the leg muscles in a positive way. The second component explained 16.75% of the variability including the a* and *h_ab_* of the LT. It was observed that meat from kids fed MR was placed to the right of that of their counterparts fed NM. Therefore, kids fed MR had legs with high values of L* and *h_ab_* and with low values of a*. On the other hand, kids fed MR had high a* value for the three muscles of the leg. Beside this effect of the RS, the breed had an influence due to the color of the LT. Therefore, Majorera, Palmera, and Tinerfeña had LTs with higher values of a* and lower values of *h_ab_* than those of the other breeds.

### 3.3 Effect of pH on muscle color

**Figure 2:**
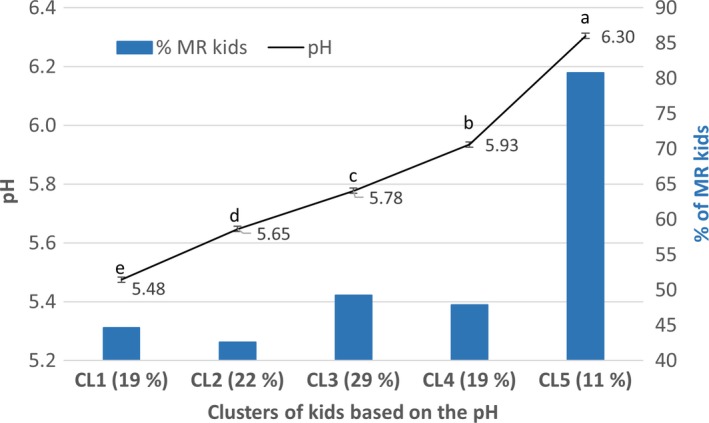
Clusters of kids based on the pH and percentage of milk replacer (MR) kids within each cluster. The percentage of kids per cluster is between brackets. The observed frequency of kids fed MR within the CL5 cluster was greater (*p* < 0.05) than the expected frequency (χ^2^ = 9.48; *p* < 0.05). Different superscripts indicate significant differences in pH between clusters (*p* < 0.05)

**Figure 3:**
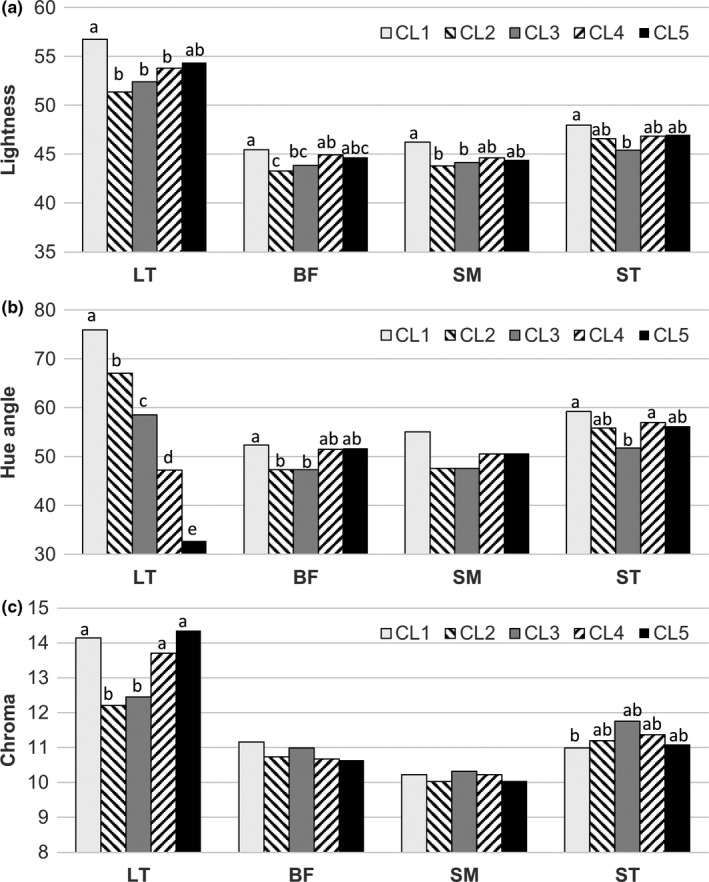
Influence of clusters based on the meat pH on the color of four muscles. BF: *biceps femoris*; LT: *longissimus thoracis*; SM: *semimembranosus*; ST: *semitendinosus*. CL1, cluster with a mean pH = 5.48; CL2, cluster with a mean pH = 5.65; CL3, cluster with a mean pH = 5.78; CL4, cluster with a mean pH = 5.93; CL5, cluster with a mean pH = 6.30. Different superscripts within a muscle indicate significant differences between clusters (*p* < 0.05)

Once the meat samples were clustered according to their pH, the percentage of samples within each cluster, average pH, and percentage of kids fed MR within each cluster were calculated and are shown in Figure 2. There were significant differences in pH between clusters (*p* < 0.001). There was a relationship between the RS and cluster of pH (χ^2^ = 11.7; *p* = 0.02). Therefore, the frequency of kids from both RSs was similar in clusters from CL1 to CL4, which had pH values below 6, but 80.8% of kids from cluster 5 were MR. The differences of the L*, *h_ab_*, and $C_{\mathrm{ab}}^{*}$ values of the four studied muscles between clusters are shown on Figure 3. The LT had a higher L* value than that of the other muscles. This muscle had a higher L* value of CL1 than those of CL2, CL3, and CL4 (*p* < 0.05) and similar value to that of CL5 (*p* > 0.05). The other muscles had moderate differences among clusters, and CL2 and CL3 tended to have lower values than CL1 and CL5. The *h_ab_* value of LT decreased almost linearly with the increase of pH, with significant differences among clusters (*p* < 0.05). However, BF, SM, and ST had a pattern similar to L*. The BF of CL1 had a higher *h_ab_* value than that of CL2 and CL3 (*p* < 0.05) and a similar value to that CL4 and CL5 (*p* > 0.05). The *h_ab_* value of SM was similar in every cluster (*p* > 0.05). The CL3 of ST had the lowest values, while the other clusters had similar values (*p* > 0.05). CL1 of LT had the highest values of $C_{\mathrm{ab}}^{*}$ together with those CL4 and CL5 (*p* > 0.05). BF and SM were not affected by pH, and the CLI of ST had the lowest $C_{\mathrm{ab}}^{*}$ value, while the other clusters had similar values (*p* > 0.05).

## 4 DISCUSSION

### 4.1 pH of the longissimus thoracis

High pH values for suckling kids are widespread in the literature, indicating that kids are inclined to stress because the goat species is excitable (Casey & Webb, 2010) and sensitive to the stress of preslaughter management (transport, lairage, etc.) (Ripoll, Alcalde, Horcada, & Panea, 2011). The pH values found in the literature for light suckling kids are similar and even higher than those reported in the present study (Bañón et al., 2006; Marichal et al., 2003; Sañudo et al., 2012; Teixeira et al., 2011). Moreover, young kids are more susceptible to emotional stress than old ones (Sañudo et al., 2012) because younger animals are still largely dependent on their mothers (Napolitano, Marino, De Rosa, Capparelli, & Bordi, 1995). It was found in lambs that artificial feeding, associated with early weaning, adversely affects the ability of lambs to manage emotional stresses (Napolitano et al., 1995), which could explain the high pH values of kids fed milk replacers and the higher frequency of kids fed milk replacers with high pH values.

### 4.2 Instrumental color of the muscles

Meat from very light suckling kids has high L* and low a* and b* values, resulting in a lighter and duller meat (De Palo, Maggiolino, Centoducati, & Tateo, 2015; Ripoll et al., 2018,2011) than beef. This meat is paler even than that from suckling kids with heavier carcasses (Ozcan et al., 2014) and concentrate‐fed kids (Emami, Nasri, Ganjkhanlou, Zali, & Rashidi, 2015). Some authors have reported that goat meat is significantly lighter and less yellow than lamb meat (Casey & Webb, 2010). Nevertheless, comparing suckling animals, kids are also lighter but have higher b* values and lower a* values than those of lambs (Lobón, Sanz, Blanco, Ripoll, & Joy, 2017). Finally, other authors have reported a difference of color ($\Delta_{\mathrm{ab}}^{*}$) between both type of meats of 1.97, which is not visually appreciable (Babiker, El Khider, & Shafie, 1990). This result is in agreement with the $\Delta_{\mathrm{ab}}^{*}$ values of 2.2 and 3 for the just noticeable difference between two *stimuli* reported by some authors (ISO, 2004; Stokes, Fairchild, & Berns, 1992). Hence, the BF, SM, and ST muscles in our study are very similar because the $\Delta_{\mathrm{ab}}^{*}$ value ranged from 1.2 to 2.9. The LT muscle is a glycolytic muscle, while the SM muscle is an oxidative muscle (Alasnier, David‐Briand, & Gandemer, 2000). However, frozen/thawed meat could show a different behavior due to the denaturalization of sarcoplasmic proteins. Argüello et al. (2005) reported that the LTL and SM muscles of light suckling kids had similar L* and *h_ab_* values, with *h_ab_* values of approximately 45, in accordance with the values we found for the leg muscles.

The meat color of preruminants, such as suckling kids, is intensely affected by the feeding system (Ozcan et al., 2014). However, the revised literature often compares the use of natural milk and milk replacers to raise kids of only one breed. Consequently, the literature results are often contradictory because it has been demonstrated that different breeds are differentially affected by feeding with milk replacers. Hence, milk replacers do not affect the L* value of fresh kid meat, independent of the measured muscle (Bañón et al., 2006; De Palo et al., 2015; Zurita‐Herrera et al., 2013). Additionally, De Palo et al. (2015) reported that the LT of kids fed natural milk had lower b* and *h_ab_* values and no difference in the $C_{\mathrm{ab}}^{*}$ value compared to those of kids fed with milk replacers. The use of milk replacers decreased the b* value of the SM muscle (Zurita‐Herrera et al., 2013). However, as previously stated, the composition of goat milk is dependent on the management system of goats (Raynal‐Ljutovac, Lagriffoul, Paccard, Guillet, & Chilliard, 2008), which influences the color of meat, especially the a* value. Some authors have explained that meat from kids reared with natural milk has lower a* and greater *h_ab_* values because the natural milk of goats is not rich in Fe (Sañudo et al., 2012). Although grazing goats had normal levels of Se and Fe in plasma (Schweinzer et al., 2017), a higher level of these elements was found in goat kids that were fed with a milk replacer (Wittek, 2002). However, Lanza et al. (2006) did not find differences in the *h_ab_* and a* values of suckling lamb meat even though the iron content of the milk replacer was greater than that of natural milk (Rodríguez et al., 2008). Goat milk has a greater bioavailability of Fe and Se than that of cow milk (Raynal‐Ljutovac et al., 2008), a principal component of milk replacers. The *h_ab_* is related to the state of the heminic pigment, while $C_{\mathrm{ab}}^{*}$ is related to the quantity of pigment (Renerre, 1982). Therefore, an increase in Fe should influence $C_{\mathrm{ab}}^{*}$ instead of *h_ab_*. Modifications in the status of the heme pigments could be related to the antioxidant status and glutathione peroxidase activity and hence the organic Se content. The organic or inorganic form of Se supplementation of goats or added to milk replacer is very important. Hence, sodium selenite act as a heme pigment pro‐oxidant, increasing metmyoglobin formation (Zhan, Wang, Zhao, Li, & Xu, 2007), while selenium in organic forms, such as selenomethionine, is reported to be a potent antioxidant (Hamilton & Tappel, 1963). Zurita‐Herrera et al. (2013) reported that the meat of kids suckling natural milk from dams grazing in pastures had a greater a* value than that of kids fed NM and MR but reared indoors, possibly due to the intake of carotenoids from pastures (Lobón et al., 2017). Kids fed NM and MR had the same L*, a*, and b* values measured for the LTL and SM muscles (Zurita‐Herrera et al., 2013). Also in disagreement with the concept of Fe deficiency, Bañón et al. (2006) and Argüello et al. (2005) reported that the LTL of kids reared with MR had a higher *h_ab_* value and the same L* value compared to those reared with NM, but the results disagreed concerning the $C_{\mathrm{ab}}^{*}$ value. The effect of RSs has also been studied in suckling lambs, where MR animals had greater a* and lower b* values (so, lower *h_ab_*) than those of NM lambs (Osorio, Zumalacárregui, Cabeza, Figueira, & Mateo, 2008).

### 4.3 Effect of pH on muscle color

Depletion of glycogen prior to slaughter due to stress often occurs in dark meat because of the high pH. However, Kannan, Kouakou, Terrill, and Gelaye (2003) did not find that dark meat from goats was affected by transport stress, although the pH was altered. Ripoll et al. (2011) studied the effect of increasing the pH of LT on suckling kid meat over a similar interval to that used in our study. These authors reported that the $C_{\mathrm{ab}}^{*}$ value increased as the pH approached 6, but remained constant between pH 6 and pH 7, which is in agreement with our study, but they did not find an effect on the L* or *h_ab_* values. Light suckling kids fed milk replacers are often more stressed, leading to meat with a high pH. This increase of pH decreases light scattering, which allows a long light path through the meat, and myoglobin becomes fully visible (Swatland, 2004). This meat is undesirable because of the large a* and $C_{\mathrm{ab}}^{*}$ values and very low *h_ab_* value and thus has a short shelf life (Ripoll et al., 2018). Argüello et al. (2005) did not find any correlations between pH and the $C_{\mathrm{ab}}^{*}$ or *h_ab_* values, probably because the highest pH in that study was below 6. However, the reported increase of pH, from 5.48 (cluster 1) to 5.65 (cluster 2), led to great changes in the L*, $C_{\mathrm{ab}}^{*}$, and *h_ab _*values. Consequently, in agreement with Napolitano, Cifuni, Pacelli, Riviezzi, and Girolami (2002), the selection of RSs should take into account animal welfare in addition to product quality.

## 5 CONCLUSIONS

The effect of the RS on the color of the studied muscles is strongly modulated by the breed. Hence, farms should consider the selection of the breed and RS together to produce meat with the color that the consumer demands. Nevertheless, since artificial RSs comprising very early weaning might induce a high pH and dark color of muscle, two strategies of artificial rearing can be proposed. The first strategy is choosing less sensitive breeds that produce meat with a normal pH. The second strategy is restricting the suckling natural milk but minimizing the separation from mothers, for example, keeping sensory (visual, olfactory, and tactile) contact between kids and mothers.

## ETHICAL STATEMENT

No humans were used in this study. All procedures involving animals were conducted according to the guidelines of Directive 2010/63/EU on the protection of animals used for experimental and other scientific purposes (EU, 2010).

## CONFLICTS OF INTEREST

The authors declare that they do not have any conflict of interest.
